# Experiments on Pilot-Scale Constructed Floating Wetlands Efficiency in Removing Agrochemicals

**DOI:** 10.3390/toxics10120790

**Published:** 2022-12-15

**Authors:** George Pavlidis, Ioanna Zotou, Helen Karasali, Anna Marousopoulou, Georgios Bariamis, Ioannis Nalbantis, Vassilios A. Tsihrintzis

**Affiliations:** 1 Centre for the Assessment of Natural Hazards and Proactive Planning & Laboratory of Reclamation Works and Water Resources Management, School of Rural, Surveying and Geoinformatics Engineering, National Technical University of Athens, 9 Heroon Polytechniou St., Zographou, 15780 Athens, Greece; 2 Laboratory of Chemical Control of Pesticides, Department of Pesticides Control and Phytopharmacy, Benaki Phytopathological Institute, 8 Stef. Delta St., Kifissia, 14561 Athens, Greece; 3 Department of Water Resources and Environmental Engineering, School of Civil Engineering, National Technical University of Athens, Greece, 9 Heroon Polytechniou St., Zographou, 15780 Athens, Greece

**Keywords:** constructed floating wetlands, agricultural wastewater treatment, nitrogen, phosphorus, pesticides, removal

## Abstract

The efficiency of constructed floating wetlands (CFWs) in their ability to remove agrochemicals (nutrients and pesticides) is here investigated in a series of pilot-scale systems. Four experimental CFWs were designed and constructed; three of them were planted with the aquatic plant species *Lemna minor*, *Azolla pinnata* and *Eichhornia crassipes*. The fourth did not contain any plants and was used as the control. The aim of the study was to evaluate the efficiency of CFW containing aquatic macrophytes in the reduction of pesticides and nutrients, under field conditions. The CFWs operated continuously from May 2021 to September 2021, and their removal efficiencies of nitrogen and phosphorus ions, and five commonly used pesticides were examined. The CFW systems were fed daily with agricultural wastewater which was prepared by mixing a fertilizer and predetermined doses of pesticides. The hydraulic residence time was kept at 14 days. Samples were collected on a weekly basis from both the influent and the effluent of each experimental tank, and were subsequently analyzed in the laboratory. HPLC-DAD and Ion Chromatography were implemented for sample analysis following a very simple sample preparation. Reductions for nutrient ranged from no reduction to 100% removal, whereas for pesticides these varied from no reduction to 98.8% removal, indicating that these systems can be used as efficient and low-cost pollution control technologies for agrochemical wastewater treatment. Significant reduction for certain pesticides was also observed in the algae control tank, thus, proving the efficiency of algae in organic pollution reduction, and recognizing the limitations of aquatic plant use in decontamination.

## 1. Introduction

During the 20th and 21st century, natural water bodies have been subjected to severe environmental pressure that resulted from both natural and anthropogenic causes [1]. The major reasons for ecosystem impairment are population growth, urbanization and intensification of industry and agriculture [2]. The use of agrochemicals plays a significant role in agricultural non-point source pollution of water bodies, posing a serious danger to drinking water resources and aquatic ecosystems. Therefore, agrochemical pollution has become a key issue of concern for the scientific community, NGOs and governments worldwide. To abate pollution, proper on-site management measures and/or use of simple and low-cost treatment technologies are required [3]. Conventional techniques designed for the treatment of wastewaters (e.g., ion exchange, adsorption, reverse osmosis, chemical precipitation, electrochemical treatment) often fail to completely remove various kinds of water contaminants; furthermore, they are expensive, energy-intensive and non-eco-friendly. An increasing need thus arises for adopting cost-effective and environmentally-friendly purification technologies for removing pollutants from water and for restoring aquatic ecosystems [4,5,6,7]. Phytoremediation is such a technology, which makes use of aquatic plants and the potential of the latter to absorb and accumulate nutrients or other substances in their tissues, thus remediating wastewater [8]. Through this process, organic and inorganic pollutants are transferred through the root system to the upper part of the plant, thus naturally purifying the contaminated soil or water [9].

Several techniques for mitigation of agricultural pollution have been proposed, including, among others, natural pollution abatement systems, such as constructed wetlands, stabilization ponds, algae systems, and agroforestry systems [10,11]. Constructed wetlands (CWs) are natural treatment systems wherein remediation of contaminated water is implemented through the physical, chemical and biological processes naturally occurring during the interaction of water and/or soil, plants and microorganisms [12]. The effectiveness of the aforementioned systems, especially when combined with aquatic macrophytes, has been well-documented for different kinds of wastewater, such as industrial, municipal, agricultural, mine waste and stormwater [5,13,14,15,16,17,18,19,20]. Various system types can be used in the treatment of the vast majority of pollutants, including organic substances, fecal pollutants, metals, nitrogen and phosphorus, pesticides in all forms, as well as PAHs, PCBs, PCEs, BTEX compounds and hydrocarbons [11,21,22,23,24,25,26,27,28,29,30,31]. A great variety of polluting substances, such as nutrients, pesticides, heavy metals, sediments and bacterial contaminants have been investigated and proven to be efficiently removed or diminished by employing aquatic macrophytes in CWs [32]. However, the effectiveness of natural pollution control systems on pesticide treatment has not been studied adequately, especially with regard to the water-based systems.

Aquatic plants established in CWs may also favor water transparency by reducing water velocity and the resultant high concentration of suspended solids in the system [20]. Although in most studies the use of CWs has had an overall beneficial effect in remediating effluents from agricultural activity, results in terms of pollutant removal effectiveness greatly vary as a result of differing environmental and design factors [33], such as climate, hydraulic loading rate, pollutant loading rate, hydraulic retention time, growth media, water depth, vegetation type and age, and percent of vegetation coverage [32]. Díaz et al. [32] reported that CWs performance in removing nitrate from agricultural runoff varies from “negative”, namely being itself a nitrate source, to 98%. Regarding total phosphorus, CWs efficiency has been found to range between non-significant to as high as 80%, whereas pesticides can be removed from agricultural effluents by 0 to 100%.

Aquatic macrophytes being exploited in CWs are subdivided into three general categories, that is, free-floating (e.g., *Pistia stratiotes, Azolla pinnata, Eichhornia crassipes, Lemna* spp.), submerged (e.g., *Myriophyllum aquaticum*) and emergent (e.g., *Typha* spp. and *Phragmites*), with plant species of the third type being the most commonly utilized in phytoremediation applications, due to their greater availability, high growth rate and ease in terms of harvesting and stocking [5,8,34]. Constructed floating wetlands (CFWs), also referred to as artificial floating islands (AFI), are a variant of CWs employing floating vegetation to remediate water [7].

As regards the aquatic plants used in the present study, *L. minor* is a free-floating aquatic species, commonly investigated for its potential in assimilating nutrients (nitrogen and phosphorus) from various sources effluents [2]. Priya et al. [35] evaluated the efficiency of *Lemna minor* in treating organic waste and removing nutrients from domestic wastewater. The experiment was conducted in a pilot scale apparatus, after primary and secondary treatment of the wastewater. The results showed good performance of the aquatic plant, which reduced BOD and orthophosphate concentrations by 94.45% and 79.39%, respectively. Aziz et al. [36] comparatively assessed the performance of four different aquatic plants, among which was *Lemna minor*, and found that the latter was capable of reducing ammoniacal nitrogen (NH_4_^+^-N) from sewage by 80.4% after eight days of treatment. Sarkheil and Safari [37] demonstrated the potential of using duckweed in aquaculture as well. In their experiment, undertaken in a recirculating water system used to culture African cichlid, *L. minor* reduced the concentrations in total nitrogen ammonia and total phosphorus by 43.7% and 52.38%, respectively [37]. Liu et al. [38] investigated the efficiency of *L. minor* in removing nitrogen (N) and phosphorus (P) from industrial wastewater, in relation to the salinity level in water. Their findings showed that high salt stress combined with long periods of exposure inhibited duckweed capacity to absorb N and P, while for NaCl concentrations above 100 mM the aquatic plant had negative removal efficiency, functioning as a sink of N and P. Ceschin et al. [39] examined the phytoremediation performance of Lemna in a three-pool CW designed for treating municipal wastewater produced from the town of Forano in Central Italy. The results highlighted the adverse effect of Lemna overgrowth on treatment system efficiency. The authors stated that the successful application of duckweed in phytoremediation requires periodic harvesting, in order to avoid the development of an extended and thick mat, which in turn impedes light penetration and favors anaerobic conditions. Kamyab et al. [40] attempted to evaluate the capacity of duckweed and microalgae to remove nutrients from palm oil mill effluents as well as to further utilize the aquatic plants for fertilizer production. The removal rate achieved from the combined use of the two species attained 12.5%, 11.3% and 70.5% for NO_3_^−^-N, NH_4_^+^-N and PO_4_^3−^-P [40]. In a similar experiment, Sudiarto et al. [41] compared Lemna with three additional aquatic plant species in terms of their ability to remove nutrients from treated livestock wastewater. The results demonstrated that duckweed was the most effective among the examined species in phosphorus uptake, achieving a removal rate of 36.15%. However, it was found to be unsuitable for nitrogen removal because of its low growth rate. Duckweed has also been successfully applied for the uptake of pesticides from agricultural runoff. Dosnon–Olette et al. [42], in a four-day experiment, tested the efficiency of Lemna along with four additional macrophyte species in removing dimethomorph and pyrimethanil, focusing also on the toxicity these substances exert on the aquatic plants depending on concentration levels. *Lemna minor* along with *S. polyrhiza* were found to be the most effective in fungicide removal with the former yielding a removal rate equal to 12% and 17% for pyrimethanil and dimethomorph, respectively. The authors also suggested that a concentration of 600 μg L^−1^ for these fungicides is suitable, so as to not inhibit photosynthetic activity of the utilized macrophytes. In a similar study conducted by Dosnon–Olette et al. [43], *L. minor* exhibited again the highest performance among the examined species in uptaking copper sulphate, flazasulfuron and dimethomorph. In this 7-day experiment, Lemna achieved a removal rate of 50%, 11.5% and 42% for copper sulphate, dimethomorph and flazasulfuron, respectively. A concentration of 40 μg L^−1^ for copper and fluzasulfuron and 400 μg L^−1^ for dimethomorph were defined as the optimum ones for evaluating remediation efficiency of the examined plants based on the toxicity test undertaken. Dosnon–Olette et al. [44] also tested *L. minor* against *S. polyrhiza*, in terms of their removal efficiency and toxicity, solely considering dimethomorph. Lemna outgrew *S. polyhriza*, reaching a removal rate of 41 μg g^−1^ of dimethomorph, for a 600 μg L^−1^ concentration after 4 days of exposure. The authors also reported a strong positive relationship between initial population density and dimethomorph toxicity for both species. The pesticide removal efficiency of *L. minor* has also been examined against chlorpyrifos by Prasertsup and Ariyakanon [45]. The experiment was undertaken under laboratory greenhouse conditions and the results showed a considerable inhibition of the relative growth rate of the aquatic plant for chlorpyrifos concentration as high as 1000 μg L^−1^, whereas the maximum removal yield was observed for 500 μg L^−1^ of the examined insecticide, reaching 87% [45]. A lower removal yield was determined for two other herbicides, namely isoproturon and glyphosate, according to an experiment conducted by Dosnon–Olette et al. [46], where *Lemna minor* was able to uptake 25% and 8% of each of the two agrochemicals, respectively, after a 4-day exposure. Furthermore, several studies have examined duckweed response in terms of removal rate and sensitivity for a variety of additional pesticides (either alone or in mixture), including metolachlor, atrazine, metribuzin, lactofen, linuron, monolinuron, diuron, 2,4-D, alachlor, paraquat, propanil, among others [47,48,49,50,51,52].

*Eichhornia crassipes*, commonly known as water hyacinth (WH), which was also used in the present study experiments, is another example of free-floating macrophyte, well-known for its pollutant removal capacity. Its effectiveness in wastewater treatment, mainly due to the assimilation of nutrients and heavy metals, has been proved by several studies [8,16]. Fox et al. [53] examined WH efficiency in removing nitrogen under different concentrations ranging between 0 and 300 ppm. The results demonstrated a 60–85% nitrogen assimilation rate after a 4-week period, whereas it was also found that biomass production, although having a positive relationship with applied nitrogen rates, stops increasing for N concentrations above 80 ppm [53]. Nabi et al. [54] elaborated on a 30-day experiment to test WH nutrient performance for domestic wastewater. The results indicated that the aquatic plant was capable of removing 63.28% and 58.54% of Total Nitrogen (TN) and phosphorus, respectively. Osti et al. [55] employed WH to improve water quality in tilapia fishponds by reducing nitrogen and phosphorus. The results revealed a reduction in TN, Total Inorganic Nitrogen (TIN), Total Phosphorus (TP) and PO_4_^3−^-P, induced by WH, equal to 66%, 82%, 27% and 33%, respectively. A 24-day experiment was performed by Kutty et al. [56] in order to assess *Eichhornia crassipes* nutrient accumulation capacity from sewage treatment plant effluent. The aquatic macrophyte exhibited a removal yield of 81%, 67% and 92% for NH_3_-N, P and NO_3_^−^-N, respectively, whereas it also presented a considerable growth rate from the sixth day and until the end of the experiment. Additional studies having investigated removal efficiency and/or sensitivity of WH in nutrient-rich wastewater are those of Sooknah and Wilkie (2004), Chen et al. (2010), Aremu et al. (2012), Zhao et al. (2012), Wang et al. (2013) and Lima et al. (2018) [57,58,59,60,61,62], among others. Despite the great availability of experiments undertaken in order to assess nutrient removal efficiency of WH, only a few have investigated WH potential for removing pesticides from agricultural wastewater. Xia and Ma [63] examined ethion uptake capacity of WH by employing four different culture solutions, that is, non-sterile planted, sterile planted, non-sterile unplanted and sterile unplanted treatment. They found that WH accounted for 69% of the total removal of the utilized ethion after 240 h of incubation against 12% of removal caused by bacterial degradation [63], with the removal rate constant of ethion due to the aquatic plant estimated at 0.00730 h^−1^. The removal capacity of *Eichhornia crassipes* against the organophosphate insecticide chlorpyrifos was investigated by Anudechakul at al. [64]. WH, along with the synergistic action of the bacterium *Acinetobacter* sp. strain WHA, achieved a removal rate constant 3.89–4.87 times higher (depending on chlorpyrifos applied concentrations) than that which occurred in the absence of plants [64]. Alencar et al. [65] comparatively examined *Pistia stratiotes* and water hyacinth regarding their efficiency in uptaking clomazone. WH was found to be more resistant in the presence of the examined herbicide, achieving, however, a lower removal yield compared to *P. stratiotes* (90 and 99.9% for WH and *P. stratiotes*, respectively) [65].

Finally, *Azolla pinnata* that was also examined in the present study, was previously investigated for its efficiency in treating four different wastewaters (domestic, municipal aquaculture and industrial) for agricultural re-use, where it recorded a complete removal of phosphorus and nitrogen compounds for all waste types tested, except for the municipal where a maximum of 75.7% was obtained for phosphorus [66]. Following the same rationale, Soman et al. [67] examined the ability of Azolla plants to remove nutrients from secondary treated wastewater and observed removals of 54.8% for ammonia, 50% for organic carbon, 71.4% for nitrites, 80.5% for total phosphorus, 91.7% for BOD and 87.4% for COD. The above findings were also supported by the study of Muvea et al. [68], as from their analysis it was observed that Azolla plants in a CFW with 10–14 days retention time efficiently removed from wastewater approx. 75% of nitrites, 32% of nitrates, 17% of ammonium and 50% of total phosphorus. However, these authors pointed out that a longer retention time would improve reductions. Another species of Azolla, the *Azolla filiculoides*, has also presented nitrogen, phosphorus and COD removals from secondary effluents treated for 28 days of up to 36%, 44% and 98.8%, respectively, thus posing that Azolla may be one of the most promising floating plants for CFW [69]. Finally, Akinbile et al. [66] reported metal removal in addition to nutrients, solids and turbidity, with removal rates reaching 70% for zinc, 99.6% for iron and 64% for magnesium.

The literature survey undertaken revealed that some work had been carried out on the potential of *Lemna minor*, *Azolla pinnata* and *Eichhornia crassipes* for removing nutrients from wastewater. Fewer studies, though, have comparatively examined the capacity of these species regarding pesticides uptake. Besides, to the best of our knowledge, there is no study having investigated the effectiveness of these macrophytes against a combination of nutrients and pesticides, which often co-exist in agricultural runoff. Therefore, the present study seeks to comparatively assess the removal efficiency of *Lemna minor*, *Azolla pinnata* and *Eichhornia crassipes*, at field-like conditions and pilot-scale systems, considering ammonium, phosphate, and nitrate ions from fertilizers, as well as the following five pesticides: (a) imidacloprid; (b) thiacloprid; (c) dimethomorph, (d) myclobutanil; and (e) difenoconazole. In order to quantify the removal rates achieved by CFW systems, the three plant species were established in respective pilot-scale CFWs continuously operating for a 16-week period, that is, from May to September 2021, whereas a fourth no-plant system, acting as control, was also established to test agrochemical removal in the absence of plants, induced though by other factors, such as hydrolysis, photolysis, bacterial and/or naturally developed algae degradation. The ultimate goal of the present experiment was to propose CFW systems for treating agricultural runoffs as well as fertilizer and pesticide residues from spraying equipment tanks, instead of discharging them, untreated, to surface water bodies. The novelty of the study lies on the fact that it provides information regarding pesticide degradation in CFW systems, where the current available literature is scarce. The paper provides new experimental data on the design and operation of these systems by examining three different aquatic macrophytes and a control algal system in parallel experiments under the same climatic conditions. A comparison is also made between warm and cold seasons accounting for the effect of temperature. Additionally, the experiment is conducted under actual field conditions in pilot-scale systems located in the open air and not in a closed laboratory. Thus, the effect of meteorological parameters can be fully examined.

## 2. Materials and Methods

### 2.1. Experimental Parameters

**Figure 1:**
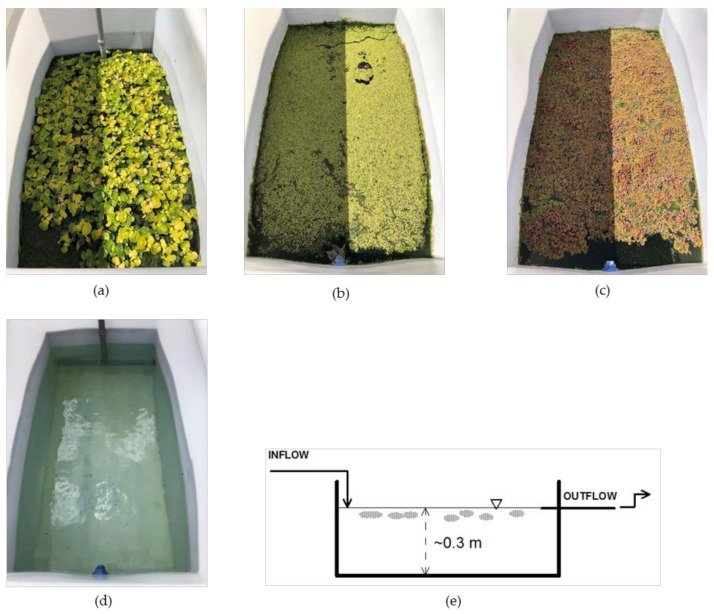
View of the main components of the experiment: (**a**) water hyacinth tank; (**b**) *Lemna minor* tank; (**c**) azola tank (**d**) control tank; (**e**) schematic representation of tanks.

The study was conducted at the premises of the School of Rural Surveying and Geoinformatics Engineering at the National Technical University of Athens Campus located in Zographou, Attica, Greece (coordinates: 37°58′30.9″ N; 23°46′47.2″ E). The tanks used for the experiment were made of hard PVC plastic, with dimensions 1.40 × 0.75 m, and were placed on the roof of the building (Figure 1). The sides of the tank were covered by black plastic material to avoid below-water sunlight impact. The study was initiated on 1 May 2021 with the necessary plant growth procedures. Wastewater loading began on 14 May and continued until 3 September 2021. The artificial waste was prepared daily right before feeding, using tap water, commercial pesticides (Plant Protection Products-PPPs) and a water-soluble 31-11-11+TE (N-P-K+micronutrients) granular fertilizer. The exact quantities of the artificial wastewater input per tank were: for imidacloprid (20% *w/v*) 0.67 mL, for thiacloprid (24% *w/v*) 0.56 mL, for myclobutanil (12% *w/v*) 1.11 mL, for difenoconazole (25% *w/v*) 0.53 mL, for dimethomorph (50% *w/v*) 0.27 mL and for the fertilizer 6.45 g, thus reaching an influent pollutant concentration of 2 mg/L for the pesticides, 30 mg/L for TN and 10.65 mg/L for TP.

Influent and effluent samples were collected on a weekly basis to determine their concentrations and estimate pollutant reductions after treatment in the systems. To eliminate any degradation, samples were deep-frozen and stored in the dark until the time of sample preparation and analysis. Wastewater loading began 2 weeks after the installation of aquatic plants to allow for aquatic plant growth and multiplication, whereas the first sampling was done one week later. The water depth in each tank was maintained at about 30 cm using an overflow weir at the tank outlet (Figure 1e) and the volume for each tank was 305 L. Wastewater feeding was performed once a day for 4 days per week at equal doses using an inverse T-shaped PVC pipe with 16 2-mm holes in its horizontal arms that was installed at the inflow side of each tank [2]. The inlet wastewater volume loaded each time was 38.1 L, thus reaching a hydraulic retention time (HRT) of 14 days, also initially implemented for plant equilibration. Apart from the chemical parameters examined, several water quality parameters were also monitored (temperature, pH, Electrical Conductivity, Total Dissolved Solids, Salinity and water depth). Finally, a meteorological station positioned next to the experimental tanks recorded basic meteorological parameters (i.e., air temperature, relative air humidity, rainfall depth, wind speed and solar radiation) at an hourly time-step with the purpose of checking possible correlations between pollutant removal efficiency and meteorological conditions.

In total, four similar tanks were used for the present experiment (Figure 1). The *L. minor* and *A. pinnata* plants used were obtained from a local nursery (Attica, Greece) and reproduced in the actual test tanks, and *Eichhornia crassipes* was available from previous experiments and was reproduced in order to be used for the present study. In parallel to the plant-containing tanks, a control tank without plants was run (however, algae developed with time, acting as an oxidation pond) and was treated the exact same way as the macrophyte-containing tanks.

Algae were eventually developed in the other three experimental tanks; therefore, the comparison with the control tank to see the effects of the two plant species was possible.

The active ingredients selected were among the most commonly used in the EU region and in Greece, and are important for agricultural production, whilst they present significantly different physicochemical and environmental fate properties (Table 1).

### 2.2. Sample Preparation

Nutrient residues sample preparation included homogeneous mixing of the sample in a table shaker at 160 rpm and filtration via a 0.45 μm and a 0.22 μm syringe filter. Appropriate dilution (1:10) was performed for the inlet samples due to their high concentration for the IC technique.

For pesticide residue determination, a filtration through 0.45 μm GF-PET filter was applied before analytical determination.

### 2.3. Chemicals, Analyses and Instrumentation

Certified stock solutions at a concentration of 1000 mg L^−1^, purchased from Dionex, USA, were used for nutrient determination after the appropriate dilution per ion in 18.3 MΩ ultrapure water. The mobile phases used were methanesulfonic acid and sodium carbonate-bicarbonate, also purchased from Dionex (Sunnyvale, CA, USA) and prepared in 18.3 MΩ ultrapure water. The analytical instrument used was a Dionex ICS-3000 (USA) system with IonPac AS 23 (4 × 250 mm) and CS16 (5 × 250 mm) columns with the respective column guards (Thermo, Waltham, MA, USA). Data acquisition and processing were performed using Chromeleon ver. 7 software. The method was validated and the recoveries for all analytes ranged between 84–111%, and the linearity coefficient values (r^2^) achieved from seven points were higher than 0.995, whilst the Limit of Quantification (LOQ) was dependent on the ion, that is, 0.1 mg/L for PO_4_^3−^-P, 0.05 mg/L for NH_4_^+^-N, and 5 mg/L for NO_3_^−^-N and was set as the lowest point concentration at the calibration curve.

Similarly, pesticide residues were determined using reversed-phase high-performance liquid chromatography with the diode array detection (HPLC-DAD) technique. A Nucleodur C-18 gravity 150 × 4.6 mm (5 μm) column was used for the quantitative determination of pesticide residues. Analytical standards of high purity were used for establishing linearity and linear range, and repeatability and accuracy of the analytical method. The analytical standard of imidacloprid (98.8%) was obtained from Bayer Crop Science, thiacloprid (98.8%) was obtained from Bayer Crop Science, dimethomorph (97.6%) was obtained from BASF, whereas myclobutanil (98.6%) and difenoconazole (99.9%) were obtained from Sigma Aldrich. The individual standard stock solutions were prepared after an appropriate dilution of the respective analytical standard to a final concentration of approximately 1 mg/mL.

Standard working solutions and their mixtures were prepared by independent dilutions of the stock solutions in acetonitrile. Acetonitrile (Merck, Darmstadt, Germany) and water (Fisher Scientific UK Limited, Loughborough, UK) were of HPLC grade. The mobile phase used for pesticide residues determination was ACN/0.1% Acetic acid aqueous solution 60/40 (*v/v*). Working standard solutions were freshly prepared from the individual stock solutions.

**Figure 2:**
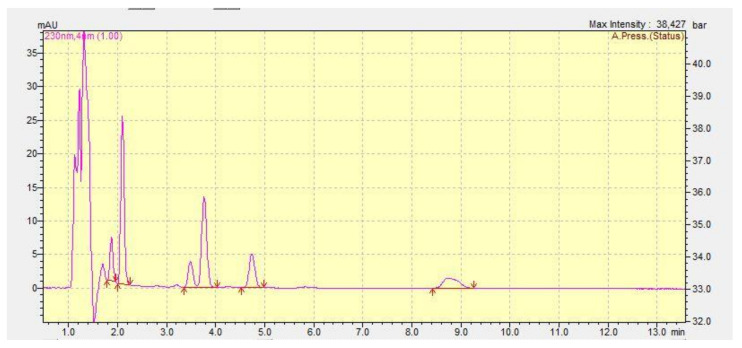
HPLC chromatogram representing the analyte peaks (as reported in Table 2).

HPLC analysis was carried out using a Shimadzu UFLC instrument (Shimadzu, Japan), equipped with a diode array detection system (SPD-M20A), a column oven (CTO-20A), a degasser (DGU-20AS) and an autosampler (SIL-20AC). The substance-specific chromatographic parameters for the examined substances are presented in Table 2. Instrument control and post-run data treatment were performed using Shimadzu Lab Solution software, version 1.25. A representative chromatogram is presented in Figure 2, whereas chromatographical method parameters are presented in Table 2. The applied methods were fully validated with respect to linearity, specificity, accuracy (in terms of recovery), and limit of detection and quantification (LOD and LOQ, respectively). The LOQ was 0.01 μg mL^−1^, for all analytes. Linearity of the chromatographic system was established using 5 calibration solutions in the range of 1 μg mL^−1^ to 50 μg mL^−1^ for the examined compounds. The correlation coefficient, as determined from the calibration curve, was 0.999 for all compounds. Recoveries ranged from 90 to 110% in all cases. Influent and effluent wastewater physicochemical parameters were measured using a portable YSI (Yellow Springs, OH, USA) Pro Plus multimeter.

### 2.4. Estimation of Pollutant Reductions

The reductions of nutrients and herbicides were calculated considering the measured concentrations in both the influent (*C_inlet_*) and the respective effluent (*C_outlet_*) for each tank, for each sampling timepoint, according to the following equation:

(1) $$\%\mathrm{Reduction}=\frac{C_{inlet}-C_{outlet}}{C_{inlet}}100$$

Mean reductions for every pollutant and system were also calculated using Equation (1) and considering the average inlet and the average outlet concentrations for the total period of monitoring.

### 2.5. Statistical Analysis

A statistical analysis was performed to investigate the significance of the derived results. The tests that were undertaken were: (a) a single-factor analysis of variance (ANOVA), in order to investigate whether the derived differences in the removal rates among the four experimental tanks are significant; (b) a Tukey’s Honest Significant Difference (HSD) post hoc test so as to identify between which particular groups (examined tanks) these differences were significant; and (c) a Student’s *t*-test in order to examine the discrepancies in pollutant removal rates per aquatic plant system, between the summer and winter period. All tests were applied in MS Excel 2016, using the data analysis toolpack and considering a significance level of 0.05. Moreover, we calculated the Pearson correlation coefficients between each recorded meteorological variable and the removal rates derived for each of the planted and the reference tank, respectively. The examined meteorological variables were the: air temperature, solar radiation, wind speed and precipitation. The above were exploited to derive estimates of potential evaporation (for the reference tank) and evapotranspiration (for the planted tanks) using the Penman (1948) and Penman–Monteith (Monteith, 1965) methods [75,76], respectively.

## 3. Results and Discussion

### 3.1. Physicochemical Parameters

**Figure 3:**
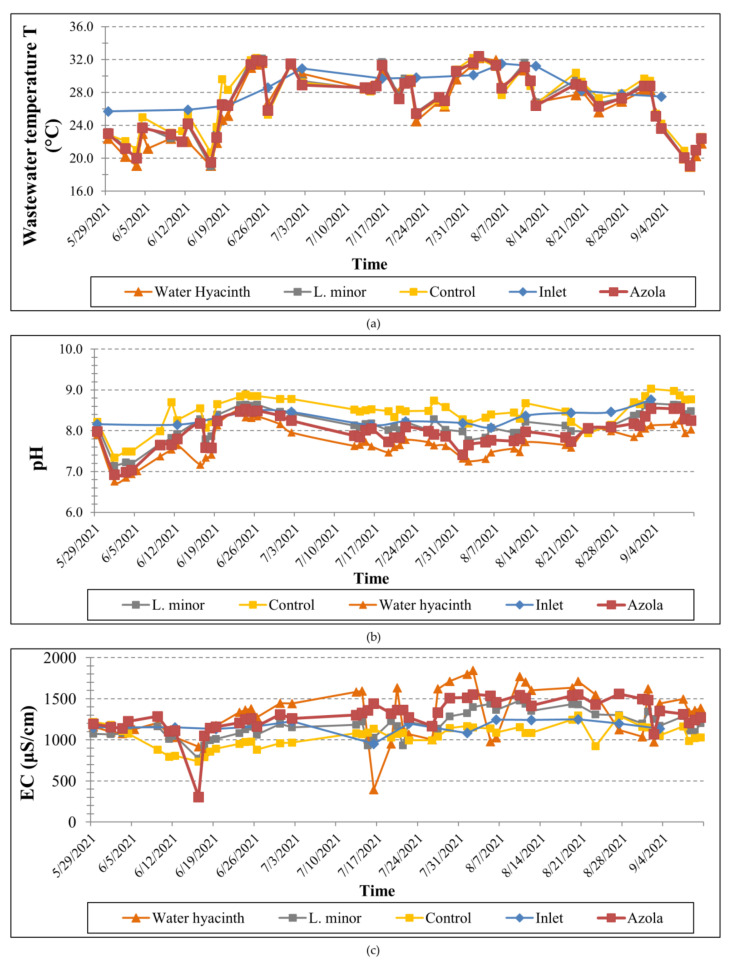
Temporal variation of (**a**) wastewater temperature, (**b**) pH and (**c**) electrical conductivity at the inlet and outlet of the four tanks throughout the operation period.

**Figure 4:**
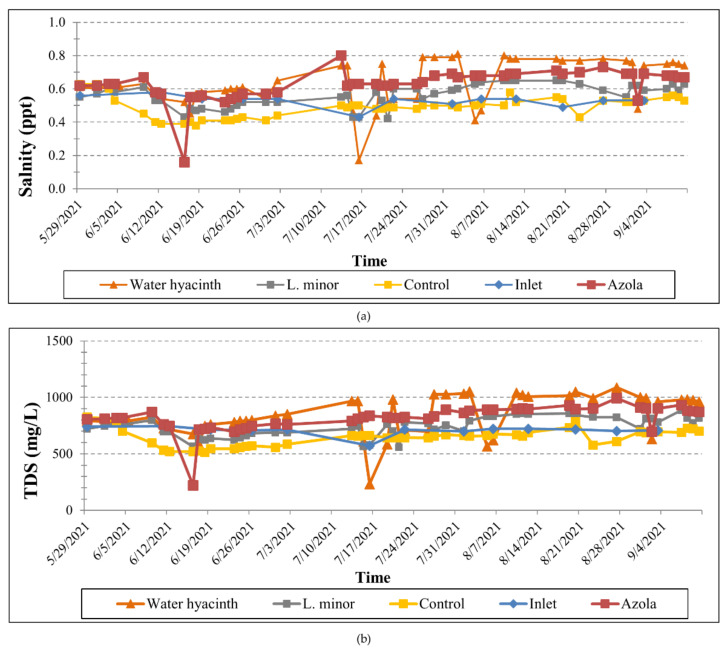
Temporal variation of (**a**) salinity and (**b**) total dissolved solids at the inlet and the outlet of the four tanks throughout the operation period.

The statistics of pH, Electrical Conductivity, Total Dissolved Solids, Salinity and water temperature (i.e., mean, standard deviation, minimum and maximum values) in the four different tanks, calculated for the entire operation period, are presented in Table 3. The temporal variation of the physicochemical parameters in the three experimental tanks, as well as at their inlet, measured during loading of the wastewater, is illustrated in Figure 3 and Figure 4.

### 3.2. Nutrients

Influent and effluent concentration and removal statistics for the overall experimental period and for all analytes were estimated and are presented in Table 4. The mean reductions were calculated considering the average influent concentration and the respective effluent levels per pollutant.

**Figure 5:**
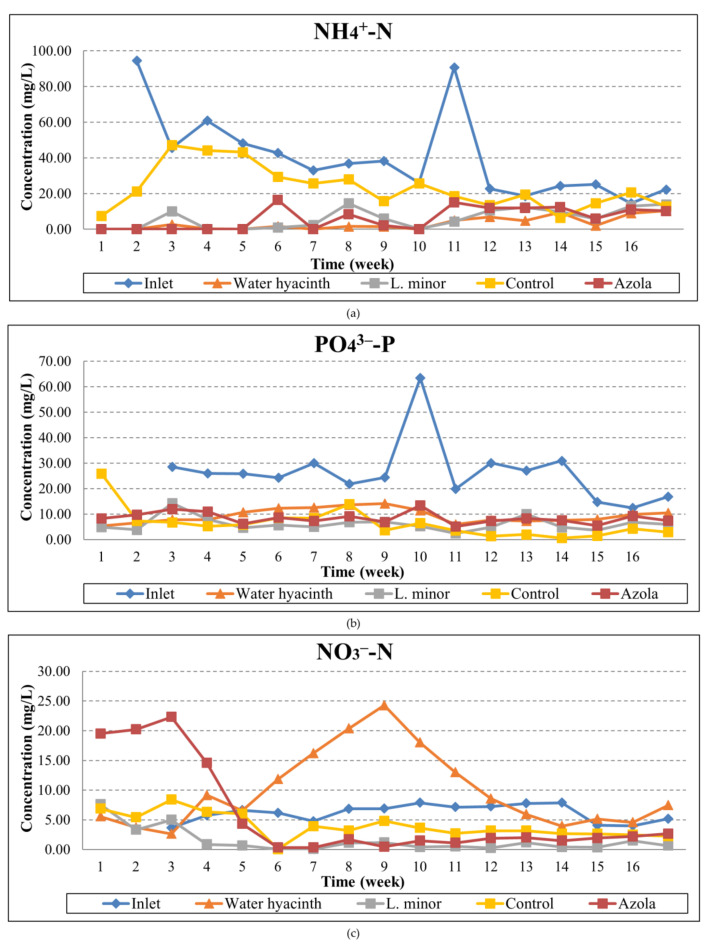
Temporal variation of: (**a**) NH_4_^+^-N, (**b**) PO_4_^3−^-P, (**c**) NO_3_^−^-N concentrations at the inlet and the outlet of the three tanks throughout the operation period.

**Figure 6:**
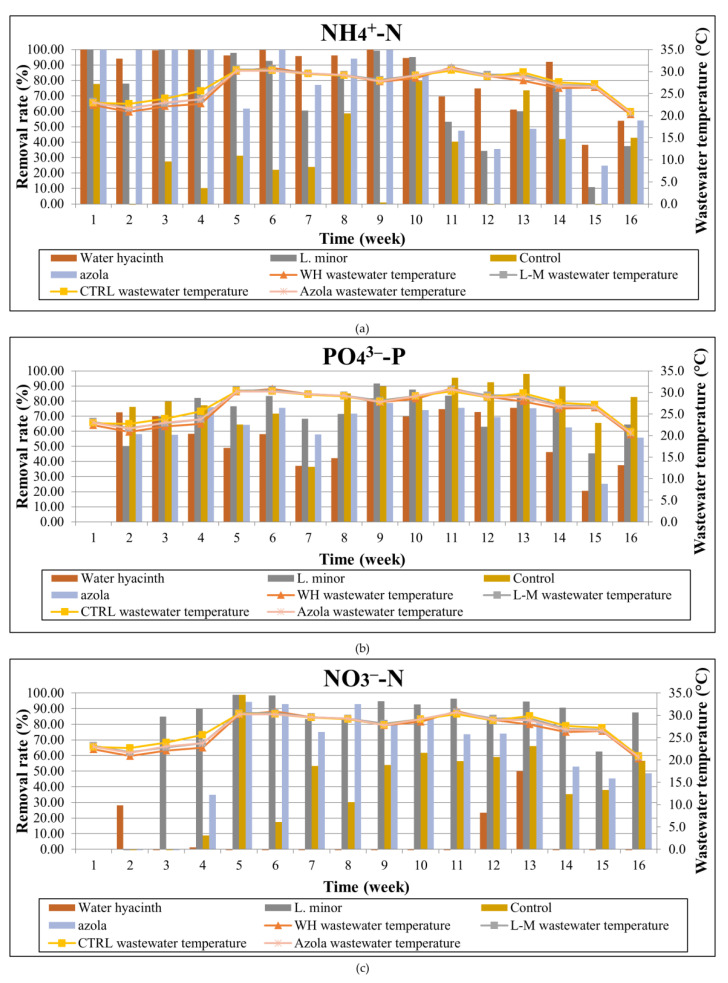
Temporal evolution of removal rate per examined tank and wastewater temperature for: (**a**) NH_4_^+^-Ν, (**b**) PO_4_^3−^-P, and (**c**) NO_3_^−^-N.

The temporal variation of ammonium, nitrate and phosphate concentrations is presented in Figure 5. Accordingly, the temporal variations of the weekly removal rates per aquatic plant and pollutant, in parallel with the wastewater temperature, are shown in Figure 6.

As can be seen from the graphs, ammonium ions exhibited reduction percentages ranging from 38.3% to 100% for water hyacinth, 11% to 100% for *L. minor*, 24.8% to 100% for *A. pinnata*, and up to 79% for the control tank. It is, therefore, noticed that the water hyacinth presented the highest reduction between the three aquatic macrophytes. The mean removal rates for the whole experiment period were thus 91.9% for water hyacinth, 84.3% for azola, 84.7% for lemna and 41.8% for the control tank.

Phosphate ions also presented significant reductions that ranged from 20.6% to 81.9% for water hyacinth, 45.5% to 91.6% for lemna, 25.3% to 78.8% for azola and between 36.6% to 98% for the algae control system, values demonstrating that the presence of algae is important for the phosphates’ uptake, as well as the fact that phosphorus is absorbed in the aquatic plants’ roots and surfaces and potentially re-dissolves during sampling and feeding procedures. The mean reductions during the 4-month experimental period ranged between 65% and 77.2%, with the minimum observed for hyacinth and the maximum for *Lemna minor*.

Finally, NO_3_^−^-N presented reductions ranging up to 50% for the water hyacinth system, up to 98.8% for the lemna system, up to 94.4% for the azola system and up to 98.9% reduction for the control tank. As a general observation, the lemna and azola systems presented a higher consistency in nitrate removal, contrary to the water hyacinth system where high variations were observed between samplings. An increase in the detected nitrate concentrations, along with an accumulation effect, was apparent from the 6th and 11th weeks of the experiment. As regards the mean reductions, they ranged from no reduction and up to 76.1%, the latter observed for the lemna system.

Our findings are generally supported by previous ones, or even presented slightly better pollution reduction potential. Comparing the present experiment performance with our previous relevant study (performed during winter; [2]) it can be concluded that reductions were comparable. In more detail, phosphate reduction was between 61.2–99.6% for lemna and 64.4–98.2% for water hyacinth, and nitrate reduction was between 18–78.4% for lemna and 19.5–78.4% for water hyacinth [2]. Using the same rationale, Sarkheil and Safari [37] observed reductions of ammonium and phosphate ions by 43.7% and 52.4%, respectively, using *Lemna minor*. In an identical combination to ours, that is, of *Lemna minor* and algae, reductions of 12.5% and 70.47% were observed for nitrates and phosphates, respectively [40], whereas Sudiarto et al. [41] presented 36.2% phosphorus uptake by lemna from livestock wastewater.

Accordingly, Fox et al. [53] reported nitrogen removals up to 85%, after a 4-week treatment using water hyacinth for N-concentrations up to 300 ppm. Similarly, Nabi et al. [54] reported water hyacinth capability for removal of 63.3% and 58.5% for TN and P, respectively, while Osti et al. [55] reported a reduction in TN, TIN, TP and PO_4_^3−^-P, equal to 66%, 82%, 27% and 33%, respectively. Significant reductions reaching 87% for nitrogen compounds and 85% for total phosphorus were also observed by Ozengin and Elmaci [77]. Wang et al. [78] reported 63.3% reduction of phosphates from a contaminated river system using water hyacinth, whilst Qin et al. [79] presented TN and TP reductions of 47.4% and 53.4%, respectively. Accordingly, Kumari and Tripathi [80] presented elimination of 26.6% of NO_3_^−^-N, 53.0% of Total Kjeldahl Nitrogen (TKN) and 56.6% of PO_4_^3−^-P concentrations.

At a different spatial scale, Wang et al. [61] studied nitrogen pollution reduction in a Chinese lake, with the results presenting reduction of 52–64% using water hyacinth. High N reductions (66–82%) and relatively lower ones for P (27–33%) were observed by Osti et al. [55] in fishponds with water hyacinth, compared to no-plant ponds, whereas in a sewage treatment study, Aremu et al. [59] reported that after a 28-day experimental period, the water hyacinth cultured sewage had reduced 45.5% of nitrate and 37.8% of phosphorus, whereas the pH also dropped from 8.6 to 7.8. Moreover, reductions of 67% of phosphorus and 92% of nitrate using water hyacinth as a treatment plant were reported by Kutty et al. [56]. Remarkable removal efficiencies of TN, P and K from wastewater that reached 63.28%, 58.54% and 85.89%, respectively, were noticed by Nabi et al. [54]. Xu et al. [81] reported removal rates of NH_4_^+^-N, NO_3_^−^-N, NO_2_^−^-N, TN, TP, COD, and Chlorophyl-a ranging between 26.4% (ultimate minimum, only for nitrites) and 99.5%. Finally, slightly lower reductions were presented by Zhao et al. in a relevant study design, where a minimum reduction of 25.6% and a maximum of 64.5% were mentioned [60].

### 3.3. Pesticides

Influent and effluent concentrations and removal statistics for the overall experimental period and for all analytes were estimated and are presented in Table 4.

**Figure 7:**
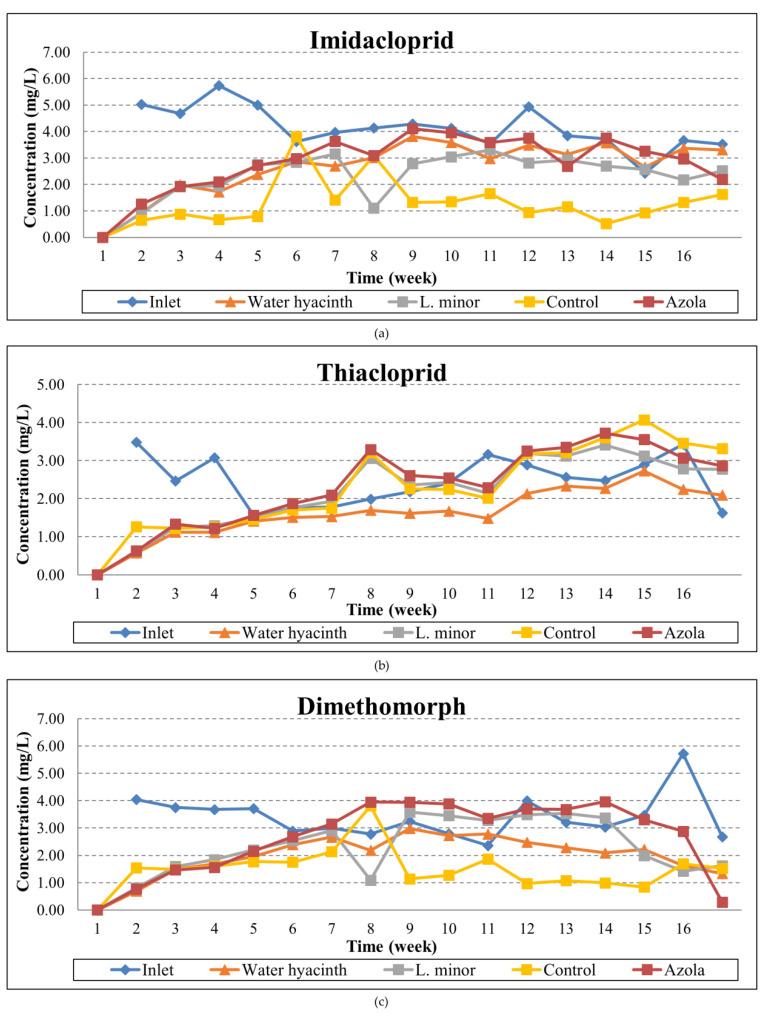

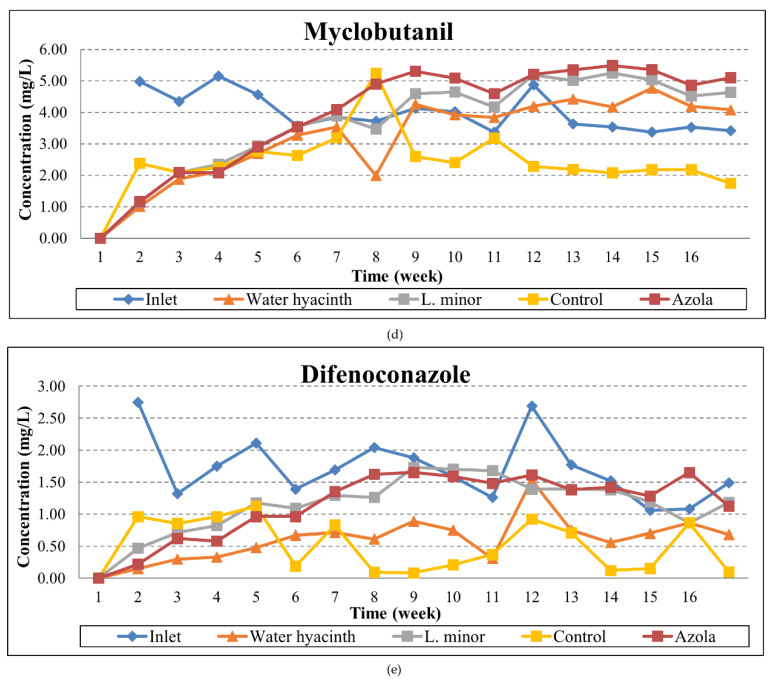
Temporal variation of: (**a**) Imidacloprid, (**b**) Thiacloprid, (**c**) Dimethomorph, (**d**) Myclobutanil and (**e**) Difenoconazole concentrations at the inlet and outlet of the three tanks throughout the operation period.

**Figure 8:**
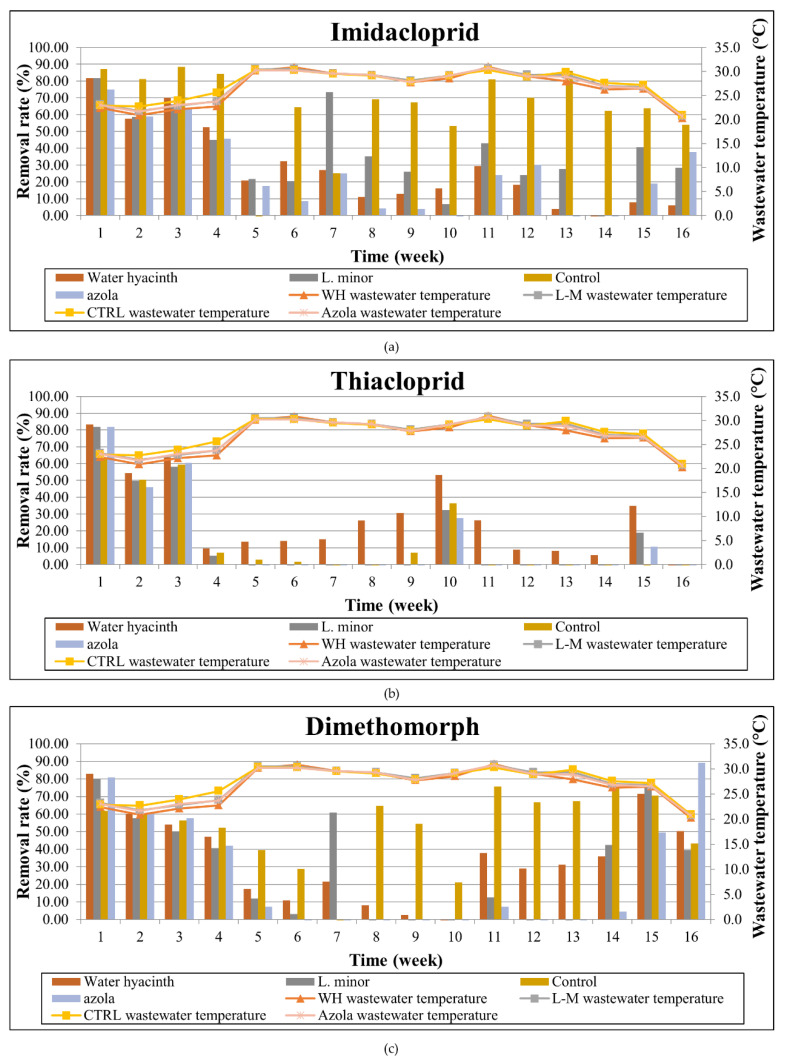

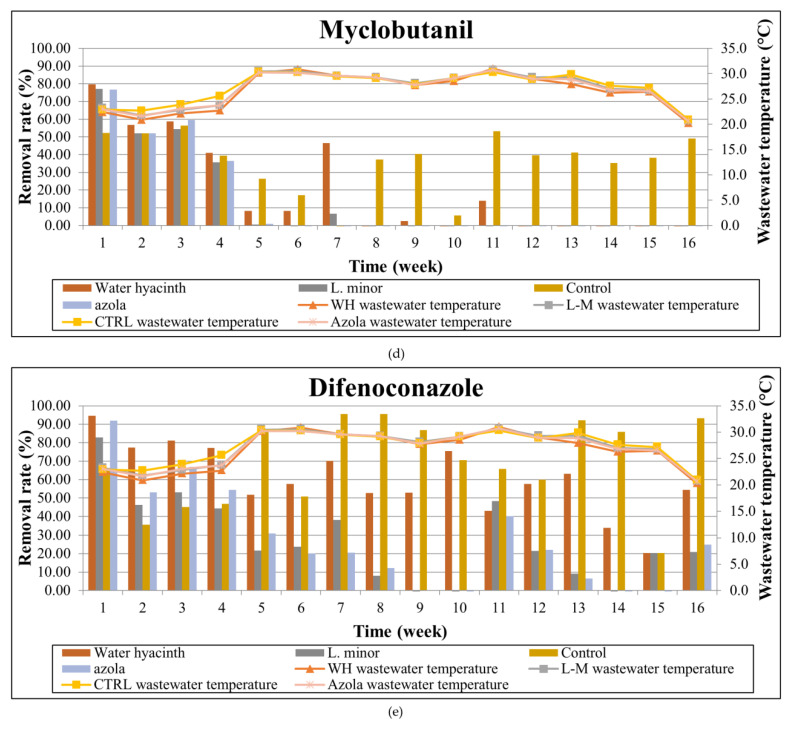
Temporal variation of removal rate per examined tank and wastewater temperature for: (**a**) Imidacloprid, (**b**) Thiacloprid, (**c**) Dimethomorph, (**d**) Myclobutanil, and (**e**) Difenoconazole.

The temporal variation and removal rates are presented in Figure 7 and Figure 8, respectively. Imidacloprid presented removals ranging from no reduction to 81.7% for water hyacinth, no removal to 81.9% for Lemna minor, no removal to 75% for Azolla pinnata and no removal to 88.3% for the algae control. Thiacloprid showed slightly better reductions in all aquatic plant systems, reaching up to 83.3% removal for the hyacinth system, 81.9% for the lemna and azola systems, and 63.8% for the algae control tank. Accordingly, dimethomorph presented reductions from no removal to 82.9% in the water hyacinth system, no removal to 80% for the lemna system, no removal to 89.2% in the azolla system and up to 75.7% for the algae control. Finally, difenoconazole exhibited disappearances up to 94.6% for the water hyacinth, 82.9% for lemna, 92% for azola and 95.6% for the algae control tank, thus proving that for this compound, the aquatic plants presence did not play a significant role, possibly due to their intrinsic environmental (DT50) or physicochemical properties (hydrolysis, photolysis).

The mean imidacloprid reductions for the whole study period were 31.1% for azola, 34.7% for water hyacinth and 43.3% for the lemna system. At the same rationale, thiacloprid was reduced by 6.6%, 34.5% and 12.6% for the respective systems, and dimethomorph reductions were 22.4%, 41.7% and 32.8% for azola, hyacinth, and lemna, respectively. Finally, as regards myclobutanil, the mean reductions were 6%, 19.5% and 7.5% for azola, hyacinth and lemna, respectively, whilst difenoconazole was respectively reduced by 33.2%, 64.8% and 33.8% throughout the experimental period.

Pesticide reductions with Lemna minor were also examined by Dosnon–Olette et al. [42], where a 17% reduction was demonstrated for dimethomorph. Similarly, dimethomorph exhibited up to 60% reduction in the study of Ekperusi et al. [82] with Lemna minor, yet with higher pesticide concentrations, reaching 1 mg/L; however, no control system was run in parallel. Additionally, in our previous experiment, under winter incubation, the reductions achieved by Lemna minor were from 10.4% to 49.9% for imidacloprid, no reduction and up to 38.8% for thiacloprid, 13.2% to 63.5% for dimethomorph and 0.8% to 60.8% for myclobutanil, with the maximum obtained for temperatures above 15 °C [2].

Regarding the degradation using water hyacinth, as reported in our previous study, the removal rates for water hyacinth treatment were from 11.4% to 65.6% for imidacloprid, no reduction and up to 57.8% for thiacloprid, 3.6% to 74.1% for dimethomorph and 4.2% to 65.1% for myclobutanil, with the maximum obtained also in this case for temperatures above 15 °C [2]. To our knowledge, there is currently no other study available in the literature considering this specific plants–pesticides combination; hence, no further comparison is feasible.

Algae that developed in the control seem to have had a positive effect in reducing pesticides, a fact that has been previously recognized in the literature [83,84,85,86]. Riaz et al. [87] also reported almost equal reductions using water hyacinth and algae for pesticide phytoremediation. Nevertheless, algae cannot be easily adopted in CFW systems as their growth is, in most cases, uncontrollable, causing system clogging and malfunctions, and at the same time creating odor and appearance issues in contrast to aquatic plant installations. In parallel, other limitations of algae systems include pH limitations, zooplankton and herbivorous protozoa contamination, need of wastewater pretreatment, and the need to establish mixed cultures to achieve maximum removal efficiency which requires special attention in order to avoid inter-species competition [84].

### 3.4. Meteorological Conditions

**Figure 9:**
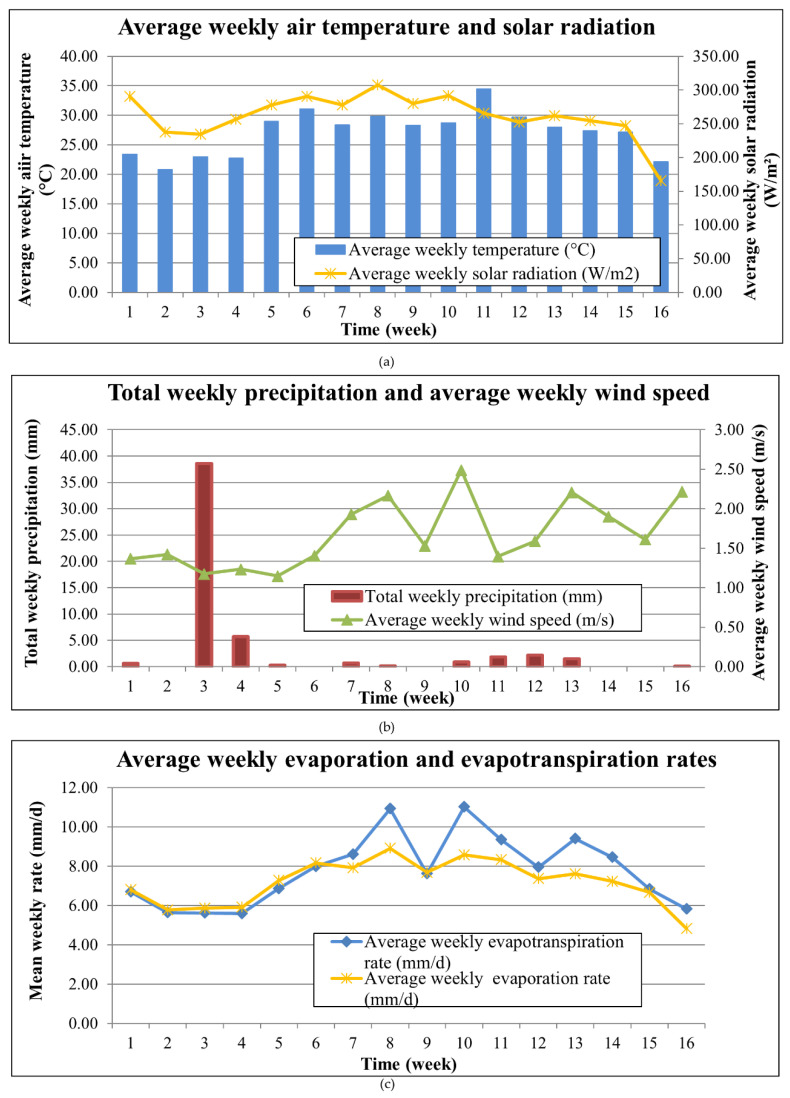
Temporal variation of (**a**) average weekly air temperature and solar radiation; (**b**) total weekly precipitation and average weekly wind speed; and (**c**) average weekly evaporation and evapotranspiration rates.

The most important meteorological parameters associated with plant growth and agrochemical decomposition, that is, air temperature, solar radiation, precipitation and wind speed, were recorded at the experimental site and are presented in Figure 9. Besides, evaporation and reference evapotranspiration rates, for the control and planted tanks, respectively, were also estimated at a daily time step, making use of the meteorological parameter records acquired from the meteorological station. The daily estimations were then averaged to give average weekly rates. The calculations were performed based on Penman and Penman–Monteith methodologies [75,76], for evaporation from open water (E_0_) and potential evapotranspiration from a reference crop (ET_0_), respectively. E_0_ and ET_0_ variation throughout the operation period are depicted in Figure 9c.

The minimum weekly average air temperature was found to be 20.8 °C and the maximum 34.5 °C. The mean daily temperature for the study period was 27.1 °C, whereas the overall minimum and maximum temperatures recorded were 14.3 °C and 42.7 °C, respectively.

### 3.5. Statistical Analysis Results

As mentioned, an ANOVA test followed by a Tukey’s Honest Significant Difference (HSD) post hoc test were implemented to investigate the statistical significance of the experimental data. To perform the ANOVA test, four groups consisting of the removal rates for each of the four experimental tanks were considered. The analysis revealed significant differences (*p ≤* 0.05) in the performance of the examined systems for almost all examined pollutants except thiacloprid. Therefore, for the rest of nutrients and herbicides, the Tukey Honest Significant Difference (HSD) post hoc test was also applied by comparing, for each pollutant, all possible pairs of groups (experimental tanks). With respect to the ammonium ions, Tukey’s test demonstrated a significantly higher performance of all aquatic plant systems compared to the algae control tank. Although water hyacinth presented relatively higher reduction rates compared to the other species, no statistically significant difference was detected among the planted tanks. Regarding phosphates, the results showed a significantly lower performance of the water hyacinth system compared to the *Lemna minor* and the algae control ones. As regards nitrates, significant differences in the reduction rates emerged between water hyacinth and *Lemna minor* and water hyacinth and control tank with the water hyacinth exhibiting a lower performance in both cases. As far as pesticides removal is concerned, the algae control tank presented a significantly better performance compared to all planted systems with respect to imidacloprid reduction, whereas in the case of dimethomorph, the latter yielded significantly higher removal rates only compared to azola. A significant difference was also identified with respect to myclobutanil removal rates between control tank and azola and *Lemna minor* aquatic systems, with the former presenting significantly higher efficiency in both cases. Finally, for the last compound, significant differences in the difenoconazole removal rates emerged between: (a) water hyacinth (higher) and azola; (b) control tank (higher) and azola; (c) water hyacinth (higher) and *L. minor*; and (d) control tank (higher) and *Lemna minor*. Table 5 presents the ANOVA results per pollutant.

Correlation coefficients between pollutant reduction rates and meteorological variables were also determined in an effort to detect eventual dependencies between the results and meteorological conditions. The Pearson coefficient values are shown in Table 6, with those revealing a strong linear relationship (r > 0.5), either positive or negative, being depicted in bold.

As a general observation, the meteorological variables that affected the efficiency of the examined systems the most are the air temperature, the evapotranspiration from the planted tanks and the wind speed, whereas precipitation did not present any significant correlation with the removal rate results, probably due to the negligible rainfall depth that was recorded during the summer period. Based on the derived correlation values, it could be concluded that the examined herbicides seem to present a more consistent behavior, that is, a negative correlation with the examined meteorological variables for most of the compounds and examined tanks. Some exceptions that are observed (positive correlation values), mainly for the cases of thiacloprid and myclobutanil, are rather low, and thus, do not indicate strong dependencies.

On the other hand, with respect to nutrient removal, it can be seen that there is a diverse image in the way the efficiency of the systems is affected by the examined meteorological variables, with correlation values sometimes revealing a positive relationship and sometimes a negative one, depending on the considered tank and pollutant. These results are in contrast with our previous relevant study [2], performed during winter, where a consistently positive correlation had been revealed between the examined system efficiency and temperature, radiation and evapotranspiration. This disagreement may indicate, especially for temperature, that there is a certain upper temperature threshold above which the aquatic plant system performance is reduced. The main reason for this is probably the increase in the concentrations of the various substances due to increased evapotranspiration and resulting water loss.

Finally, an attempt was made to compare the pollutant removal performance of the examined systems during summer (current experiment) and winter period, when a similar experiment had been elaborated [2]. For this purpose, we considered a 2-month period, from November 2020 until early January 2021 where the average wastewater temperatures were below 15 °C using removal rates by Pavlidis et al. [2], and compared with respective data from the current experiment, undertaken during the summer season, when the wastewater in the experimental tanks had an average temperature of approximately 27 °C. To analyze the differences in the results between the two experiments, we performed a two-sample Student’s t-test by examining each combination of experimental tank and pollutant separately. It should be noted that only the compounds and experimental systems that had been considered in both experiments were included in the analysis, namely, water hyacinth, *L. minor* and the control tank with respect to the examined systems, and phosphates, nitrates, imidacloprid, thiacloprid, dimethomorph and myclobutanil with respect to the considered pollutants. The results revealed a significant difference (*p* = 0.001) between the winter and summer periods for the case of water hyacinth and phosphate and nitrate removals, indicating a significantly higher level of performance of the specific aquatic plant for both compounds during the winter. On the contrary, a significantly higher (*p* = 0.00008) removal performance with respect to nitrates was seen for *L. minor* during the summer period. Regarding pesticide reduction, the examined aquatic plant systems did not show any significant difference in their performance between summer and winter. Some significant discrepancies were only derived for the control tank and for three of the examined pesticides, that is, imidacloprid (*p* = 0.00002), dimethomorph (*p* = 0.0003), and myclobutanil (*p* = 0.01). For these three compounds, the analysis demonstrated a significantly higher efficiency of the algae control tank during the current experiment (summer season).

## 4. Conclusions

The necessity, significance, and efficiency of natural treatment and remediation systems has been previously reported in various studies dealing with domestic wastewater treatment, with regards to organic matter, nitrogen and phosphorus removal. The constructed floating wetlands (CFW) that were also examined in the present study constitute an efficient, easily applicable and low-cost treatment option. In this context, the efficiency of three different constructed floating wetlands pilot-scale systems consisting of aquatic macrophytes as a pollution control plant, in the removal of nutrients and five pesticides was examined in comparison with an unplanted system over a 16-week period during spring to summer of 2021, indicating very promising results. Reductions reaching almost 100% were found for all examined agrochemicals. The highest mean reduction percentages were observed for phosphates, ammonium, nitrates and difenoconazole.

Both for ammonium, nitrates and thiacloprid, the aquatic macrophyte systems performed better than the control. On the contrary, potassium presented high residues, possibly due to adsorption and accumulation to plant roots, with subsequent re-suspension in the water column. Moreover, for the rest of the pesticides, the reductions observed in the plant systems were comparable to those of the control system, demonstrating that the effect of aquatic plants was comparable to the one provided by the green algae which developed in the control tank. It shall also be remarked that the potential for plant uptake and absorption to aquatic plant surfaces cannot be neglected based on the pesticide physicochemical properties. An exception was observed for imidacloprid, where photolytic degradation was the prevailing dissipation process, which was rather expectable based on the photolytic half-life of the compound (1 h; Table 1).

Overall, the statistical analysis results revealed a greater dependency of the pesticides removal potential on the examined meteorological parameters, especially temperature, wind speed and evapotranspiration. In particular, an increase in these variables proved to negatively affect the aquatic plant system efficiency (negative correlation), which is in contradiction with our previously published results derived from a similar experiment running during the autumn to winter period. This may be explained by the subsequent reduction in water volume in the tanks due to the high evapotranspiration rates, and therefore, the consequent increase in pollutant concentrations. Besides, the differentiated results between the two periods demonstrate the existence of specific threshold values of the examined variables, especially air temperature, above which pesticide decontamination starts being hampered. Additional experimentation under longer and/or different periods (e.g., spring season) characterized by different temperature ranges is required to derive generalized results.

It can be concluded that the examined systems under the Mediterranean geoclimatic conditions described in the present study have the potential for decontamination of spray tank mix remnants, or pesticide container wash-off water, as well as agricultural runoff from agricultural field drainage networks, thus consisting of a valuable technology for farmers and policy-makers. Further research is deemed necessary, considering different pollutant and plant combinations, variation of HRT, analysis of aquatic macrophytes to establish mass balance, as well as the correlation of pollution reduction with chlorophyll content and other plant-related parameters.

## Figures and Tables

**Table 1:** The examined pesticides and their environmental fate endpoints [70,71,72,73,74].

Substance	NOEC (Studies with *L. gibba*)	Water Solubility (mg/L)	Koc (Adsorption) (mL/g)	DT50soil	Photolysis	Hydrolysis	DT50water	DT50 System	Type
Myclobutanil	105 mg as/L	132	225–920	191–1216 d lab/ 9–58 d field	Stable	Stable	4–20 d	415–838 d	fungicide
Imidacloprid	no data	600	109–411	27–180 d field	Yes DT50 = 1 h	Stable	>30 d	129 d	insecticide
Difenoconazole	no data	15	400–7730	20–242 d field	Stable	Stable	1–2 d	307–324 d	fungicide
Thiacloprid	46.8 mg as/L	184	393–870	6–16.8 d field	Stable (79.7 d)	Stable	n.a.	12.1–18 d	insecticide
Dimethomorph	no data	10.7–47.2	290–566	34–53.4 d lab/ 10–61 d field	Stable (86–107 d)	Stable	5–15 d	16–59 d	fungicide

**Table 2:** Chromatographical parameters.

Analyte	Detector/ Wavelength	Flow Rate (mL/min)	Injection Volume (μL)	Retention Time (min)	Column Oven Temperature (°C)
Imidacloprid	UV (230 nm)	1.0	10	1.85	35
Thiacloprid	UV (230 nm)	1.0	10	2.05	35
Dimethomorph	UV (230 nm)	1.0	10	3.67	35
Myclobutanil	UV (230 nm)	1.0	10	4.62	35
Difenoconazole	UV (230 nm)	1.0	10	8.65	35
Nitrates	El. Conductivity	1.0	1000	13.0	30
Phosphates	El. Conductivity	1.0	1000	16.8	30
Potassium	El. Conductivity	1.0	1000	5.6	30

**Table 3:** Physicochemical parameters in the test system tanks.

Statistical Parameter	T (°C)				Salinity (ppt)				pH			
	Hyac.	Azola	Lemna	Contr.	Hyac.	Azola	Lemna	Contr.	Hyac.	Azola	Lemna	Cont.
Minimum	19.0	19.1	19.0	19.0	0.2	0.2	0.4	0.4	6.8	6.9	7.1	7.3
Mean	26.3	26.8	26.9	27.1	0.6	0.6	0.6	0.5	7.7	8.0	8.1	8.5
Maximum	32.2	32.4	32.5	32.2	0.8	0.8	0.7	0.6	8.5	8.6	8.7	9.0
St. Dev.	4.01	3.81	3.91	3.66	0.14	0.09	0.07	0.06	0.40	0.39	0.37	0.38
	**EC (μS/cm)**				**TDS (mg/L)**							
	**Hyac.**	**Azola**	**Lemna**	**Contr.**	**Hyac.**	**Azola**	**Lemna**	**Contr.**				
Minimum	394.7	306.1	771.0	737.0	233.4	221.0	559.0	513.5				
Mean	1300.3	1301.5	1181.4	1044.9	840.0	818.3	740.3	648.0				
Maximum	1845.0	1560.0	1479.0	1297.0	1087.5	992.0	886.5	825.5				
St. Dev.	306.30	209.43	157.19	130.48	176.85	114.28	87.81	77.57				

**Table 4:** Removal percentages for the examined systems.

Pollutant	System			
	Azola	Water Hyacinth	Lemna	Control
**NH_4_^+^-N (%)**				
Mean	84.3	91.9	84.7	41.8
Max	100.0	100.0	100.0	79.6
Min	24.8	38.3	10.9	0.0
**PO_4_^3−^-P (%)**				
Mean	68.5	65.0	77.2	76.2
Max	78.8	81.9	91.6	98.1
Min	25.3	20.6	45.5	36.4
**NO_3_^−^-N (%)**				
Mean	6.6	20.4	76.1	35.8
Max	94.4	50.0	98.8	98.9
Min	0.0	0.0	0.0	0.0
**Imidacloprid (%)**				
Mean	31.1	34.7	43.3	68.3
Max	75.0	81.7	81.9	88.3
Min	0.0	0.0	0.0	0.0
**Thiacloprid (%)**				
Mean	6.6	34.5	12.6	0.9
Max	81.9	83.3	81.9	63.8
Min	0.0	0.0	0.0	0.0
**Dimethomorph (%)**				
Mean	22.4	41.7	32.8	53.1
Max	89.2	82.9	80.0	75.7
Min	0.0	0.0	0.0	0.0
**Myclobutanil (%)**				
Mean	6.0	19.5	7.5	34.9
Max	76.7	79.7	77.1	56.3
Min	0.0	0.0	0.0	0.0
**Difenoconazole (%)**				
Mean	33.2	64.8	33.8	69.0
Max	92.0	94.6	82.9	95.6
Min	0.0	20.40	0.0	20.4

**Table 5:** Results of ANOVA test performed between the four experimental tanks, that is, azola, water hyacinth, *Lemna minor* and control tank, for each of the examined pollutants.

Pollutant	F	*p*-Value	F Crit
NH_4_^+^-N *	12.990	1.21 × 10^−6^	2.758
PO_4_^3—^-P *	5.555	0.002	2.769
NO_3_^—^-N *	6.237	0.001	2.769
Imidacloprid *	8.370	9.82 × 10^−5^	2.758
Thiacloprid	2.196	0.098	2.758
Dimethomorph *	3.322	0.026	2.758
Myclobutanil *	4.527	0.006	2.758
Difenoconazole *	12.376	2.10 × 10^−6^	2.758

* Statistically significant difference (*p* ≤ 0.05).

**Table 6:** Pearson correlation coefficients between meteorological variables and pollutant reduction rates per pollutant and examined tank. Bold values indicate a strong linear relationship.

Pollutant								
Examined Tank	NH_4_^+^-N	PO_4_^3−^-P	NO_3_^−^-N	Imidacroprid	Thiacloprid	Dimethomorph	Myclobutanil	Difenoconazole
	Pearson Correlation Coefficients between Temperature and Pollutant Reduction							
Azola	−0.352	0.347	**0.648**	**−0.607**	−0.357	**−0.718**	**−0.511**	−0.402
Water hyacinth	−0.082	0.096	−0.364	−0.480	−0.197	**−0.591**	−0.450	−0.497
Lemna	−0.149	0.441	**0.538**	−0.408	−0.342	**−0.575**	**−0.515**	−0.356
Control	0.098	0.129	**0.594**	−0.251	−0.202	−0.044	−0.308	0.276
	**Pearson correlation coefficients between solar radiation and pollutant reduction**							
Azola	0.340	0.441	0.329	−0.289	0.250	**−0.622**	0.081	−0.049
Water hyacinth	**0.539**	0.169	**−0.545**	0.073	0.420	−0.478	0.135	0.122
Lemna	0.486	0.471	0.243	−0.013	0.289	−0.362	0.127	−0.129
Control	0.266	−0.111	0.196	−0.103	0.431	−0.163	−0.373	0.133
**Pearson correlation coefficients between evapotranspiration/evaporation and pollutant reduction**								
Azola	−0.128	0.402	**0.505**	**−0.671**	−0.281	**−0.782**	**−0.560**	−0.457
Water hyacinth	0.022	0.091	−0.368	**−0.514**	−0.038	**−0.690**	−0.456	−0.210
Lemna	−0.013	0.439	0.368	−0.411	−0.247	**−0.652**	**−0.514**	**−0.577**
Control	0.317	0.053	0.413	−0.179	0.041	−0.170	−0.445	0.350
**Pearson correlation coefficients between wind speed and pollutant reduction**								
Azola	−0.212	0.050	0.299	**−0.523**	−0.476	−0.392	**−0.692**	**−0.537**
Water hyacinth	−0.297	−0.184	−0.114	**−0.624**	−0.299	−0.422	**−0.559**	−0.131
Lemna	−0.278	0.097	0.143	−0.398	−0.454	−0.385	**−0.619**	**−0.650**
Control	0.473	0.142	0.284	−0.078	−0.496	−0.206	−0.331	**0.532**
**Pearson correlation coefficients between precipitation and pollutant reduction**								
Azola	0.238	−0.111	−0.275	0.423	0.368	0.301	0.438	0.381
Water hyacinth	0.174	0.203	0.096	0.466	0.308	0.225	0.369	0.333
Lemna	0.236	−0.047	0.071	0.335	0.334	0.191	0.398	0.305
Control	−0.020	0.039	−0.213	0.293	0.336	0.067	0.271	−0.272

## Data Availability

All data are included in the paper.
